# Assessment of myocardial infarction in mice by Late Gadolinium Enhancement MR imaging using an inversion recovery pulse sequence at 9.4T

**DOI:** 10.1186/1532-429X-10-6

**Published:** 2008-01-24

**Authors:** Catherine Chapon, Amy H Herlihy, Kishore K Bhakoo

**Affiliations:** 1 Stem Cell Imaging, MRC Clinical Sciences Centre, Hammersmith Hospital, Imperial College London, UK; 2 Biological Imaging Centre, MRC Clinical Sciences Centre, Hammersmith Hospital, Imperial College London, UK

## Abstract

**Purpose:**

To demonstrate the feasibility of using an inversion recovery pulse sequence and to define the optimal inversion time (TI) to assess myocardial infarction in mice by late gadolinium enhancement (LGE) MRI at 9.4T, and to obtain the maximal contrast between the infarcted and the viable myocardium.

**Methods:**

MRI was performed at 9.4T in mice, two days after induction of myocardial infarction (n = 4). For cardiovascular MR imaging, a segmented magnetization-prepared fast low angle shot (MP-FLASH) sequence was used with varied TIs ranging from 40 to 420 ms following administration of gadolinium-DTPA at 0.6 mmol/kg. Contrast-to-noise (CNR) and signal-to-noise ratio (SNR) were measured and compared for each myocardial region of interest (ROI).

**Results:**

The optimal TI, which corresponded to a minimum SNR in the normal myocardium, was 268 ms ± 27.3. The SNR in the viable myocardium was significantly different from that found in the infarcted myocardium (17.2 ± 2.4 vs 82.1 ± 10.8; p = 0.006) leading to a maximal relative SI (Signal Intensity) between those two areas (344.9 ± 60.4).

**Conclusion:**

Despite the rapid heart rate in mice, our study demonstrates that LGE MRI can be performed at 9.4T using a protocol similar to the one used for clinical MR diagnosis of myocardial infarction.

## Introduction

Late Gadolinium Enhancement (LGE) cardiovascular magnetic resonance (CMR) imaging using a T1-weighted sequence with an inversion recovery (IR) pre-pulse is commonly used for the clinical diagnosis of myocardial infarction in humans [1,2], and experimentally in large animals [3]. The time to inversion (TI) is selected to null the signal intensity (SI) in the non-infarcted (viable) myocardium, following administration of chelated gadolinium, in order to increase contrast between the viable and hyper-enhanced infarcted myocardium [1]. Late gadolinium enhancement MR imaging with inversion recovery techniques has been shown to correlate well as an early marker of irreversible injury and eventual fibrosis [3].

More recently, the mouse model has been used increasingly for investigating the mechanisms of myocardial diseases. Hence, its essential to assess myocardial infarction in pre-clinical models in a manner similar to that used for clinical diagnosis. At present the assessment of infarct size in small animals at high magnetic fields is performed using a 'cine gradient echo pulse sequence' with a high dose of gadolinium chelates [4]. However, this type of MR protocol is limited in its ability to accurately delineate the infarcted tissue from the normal non-infarcted myocardium [1,5]. A number of discrepancies have also been observed between different studies using LGE, due principally to the use of differing protocols, including dissimilar MR sequences for imaging acquisition, different delay periods after administration of contrast agent, and even different animal models of MI. However, the use of an inversion recovery T1-weighted approach has become an accepted modality for the clinical diagnosis of myocardial infarction [6,7]. Nevertheless, this approach has not been extensively described in the literature for cardiac MR studies in small animals at high magnetic field (such as 9.4T), due to the rapid heart rate (500–600 bpm in mice and 300–400 bpm in rats) [4].

Thus, the aims of the present study were firstly, to demonstrate the feasibility of using an inversion recovery pulse sequence to assess myocardial infarction in mice following administration of gadolinium chelate, and secondly, to define the optimal inversion time for myocardial infarction MR imaging at 9.4T in order to discriminate the infarcted tissue from the non-infarcted myocardium.

## Methods

### Animal model

All studies were performed in accordance with the Animals (Scientific Procedures) Act 1986 (UK) and local institutional guidelines. During the surgical procedure, male C57Bl/6 mice (10–12 weeks old, 25–30 g, Harlan, UK; n = 4) were anaesthetized with 1.5–2% isoflurane-oxygen mixture, intubated and ventilated using a small animal ventilation apparatus (VetTech Solutions Ltd, UK). Following thoracotomy, myocardial infarction was achieved by permanent ligation of the left coronary artery using a 7-0 silk suture tied around the left anterior descending coronary artery approximately 1–2 mm from its origin. The ischemic area was identified visually as a result of blanching of the tissue upon ligation.

### CMR imaging

Two days after myocardial infarction, cardiovascular MR imaging was performed on a 9.4T horizontal MR scanner (Varian, Palo Alto, CA, USA), using an eight rod quadrature birdcage transmit/receive coil with an internal diameter of 25 mm and a length of 25 mm (Magnetic Resonance Laboratories, Oxford, UK). The gradient strength was 20 G/cm with a rise time of 90 usec.

**Figure 1:**
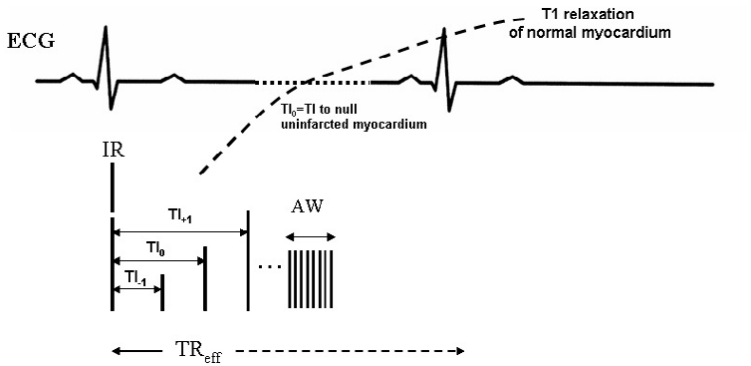
Diagram of MR imaging with inversion recovery magnetization-prepared fast low angle shot (MP-FLASH) pulse sequence. IR = inversion recovery pulse; ECG = electrocardiogram; AW = acquisition window; TI = inversion time (optimal inversion is TI_0 _= TI to null uninfarcted myocardium); TR_eff _= effective repetition time (depending on TI and heart beat).

For late gadolinium enhanced MR imaging, a 0.6 mmol/kg bolus of Gd-DTPA (Magnevist, Schering Healthcare, UK) was injected intravenously through the tail vein using a 27G catheter. ECG was recorded with a monitoring and gating system (SA Instruments, Inc. Stony Brook, NY, USA), to provide a trigger pulse at the R-wave signal. Scout Gradient echo images were acquired in order to obtain the short-axis plan of the heart. Imaging was initiated immediately after the injection of Gd-DTPA using an ECG-gated segmented magnetization-prepared FLASH sequence. The following parameters were used for image acquisition: TReff~120 ms; TE = 2.2 ms; FOV = 25 × 25 mm; matrix 128 × 128; flip angle = 30°; 20 averages; 16 segments, giving 8 phase encode steps per cardiac cycle. Thus, the data acquisition time per inversion pulse within each cardiac cycle was 18 msec, to ensure the imaging was performed in a short window centred at the null point. We used segmented k-space to decrease the total imaging time and to ensure consistent contrast across the image. By collecting 8 phase encode steps at a time we kept k-space centred around the null point of the T1 recovery. A midventricular short axis slice of 1.2 mm was selected for this study. The TI for this sequence was defined as the time between the non-selective adiabatic IR pulse and the 30° flip angle RF pulse. TI varied as follows: 20 points were obtained where TI of the first image was 40 ms, with subsequent TI applied at 20 ms intervals i.e. from 40 to 420 ms. The k-space lines were acquired in center-out interleaved linear order (Figure 1). CMR imaging was initiated immediately after contrast injection and continued for 45 minutes.

### MR image analysis

MRI images have been evaluated according to the protocol reported by Gupta *et al *[2], where the optimal TI (TI_0_) was defined visually as the value at which the signal intensity (SI) of the viable myocardium was nulled. Signal intensities were measured in 2 to 3 regions of interest (ROIs) from the most intense portion of the hyper-enhanced segments corresponding to the infarcted myocardium, 3 ROIs in blood pool in the left ventricle and 3 ROIs in the viable myocardium. The noise was measured in a region undegraded by artefacts. Signal-to-noise ratios (SNRs) for different cardiac regions were calculated by dividing each mean SI by the standard deviation (SD) of the noise. The SNRs for all TI values were plotted for each mouse, and the SNR for the optimal TI was compared with the SNR of other TIs. The contrast-to-noise ratios (CNR) for the infarcted myocardium compared with the viable myocardium was defined as (SI_inf _- SI_viable_)/SD_noise_. The relative SI (expressed as a percentage) of the infarcted myocardium compared with the viable myocardium was defined as [(SI_inf_-SI_viable_)/SI_viable_] × 100. The relative SI (expressed as a percentage) of the blood pool compared with the viable myocardium was defined as [(SI_blood_-SI_viable_)/SI_viable_] × 100.

### Post-mortem analysis

Following image acquisition, animals were euthanized. The heart was excised and sectioned into 1 mm thick slices along the short axis and the presence of myocardial infarction was confirmed by incubation of heart slices in 2,3,5-triphenyltetrazolium chloride (TTC) for 20 minutes at 37°C.

### Statistical analysis

All values were expressed as the mean plus or minus the standard error of the mean (± SEM). SNR and CNR of different regions of interest and the relative SIs (%) between regions of interest over time were compared with ANOVA of repeated measurements. Multiple comparisons were analysed by the Bonferroni method. Values of P < 0.05 were considered statistically significant.

## Results

**Figure 2:**
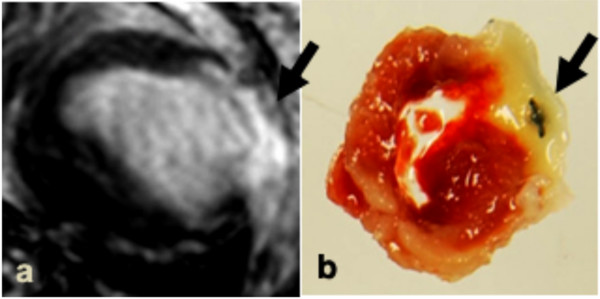
**a) **Short-axis MR images performed 45 minutes post-injection using a TI of 300 msec and **b) **corresponding post-mortem image following TTC staining. Arrows indicate the infarcted myocardium defined using MRI (a) and post-mortem TTC staining (b).

**Figure 3:**
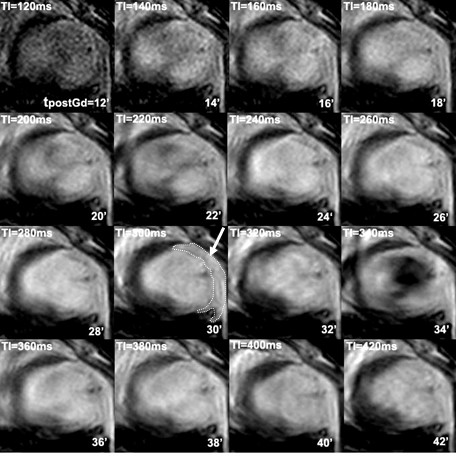
Short-axis cardiovascular MR images corresponding to each inversion time (TI) following Gd-DTPA injection (the dotted line shows the hyperenhanced area corresponding to the infarcted myocardium at 30 minutes post-Gd-DOTA injection).

**Figure 4:**
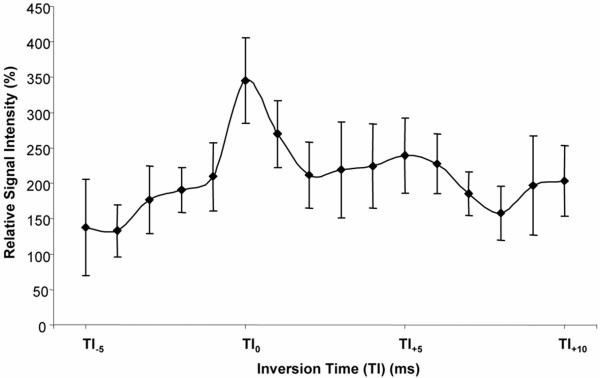
Mean relative SI (%) (± SEM) of infarcted compared with viable myocardium at varying TIs.

Transmural myocardial infarction was confirmed in all mice using TTC staining (Figure 2). The TI_0 _was defined as the TI value, which nulls the SI of the non-infarcted (viable) myocardium. This value was unique for each mouse and ranged from 220 to 300 msec (Figure 3). The SNR in the viable myocardium was at its minimum (17.2 ± 2.4, ranging from 12.8 to 23.1) for a mean TI of 268 msec ± 27.3 corresponding to the optimal TI (referred to as TI_0_). Mean SNR, CNR and relative SI at TI_0 _are reported in Table 1. The minimum SNR, at TI_0_, in the viable myocardium (17.2 ± 2.4) was significantly different from that found in the infarcted myocardium (82.1 ± 10.8; p = 0.006) but not from the SNR in the blood (48.7 ± 16.6, p = 0.126). The mean CNR of infarcted tissue compared with viable myocardium was 76.6 ± 19.8, whereas infarcted tissue compared with blood was 25.7 ± 14.7, and blood compared with viable myocardium was 40.6 ± 21.2. The mean maximum relative SI of the infarcted tissue compared with viable myocardium was 344.9 ± 60.4% (Figure 4), and occurred at TI_0 _when SI of the viable myocardium was minimum, leading to an optimal CNR between viable and infarcted myocardial areas.

**Table 1:** SNR, contrast-to-noise ratio, and relative SI values in different regions of interest in myocardium at TI_0_.

**a) SNR:**	
Normal myocardium	17.2 ± 2.4*
Infarcted myocardium	82.1 ± 10.8
Blood	48.7 ± 16.6
**b) Contrast-to-noise ratio:**	
Infarcted compared with normal myocardium	76.6 ± 19.8
Infarcted compared with blood	25.7 ± 14.7
Blood compared with normal myocardium	40.6 ± 21.2
**c) Relative SI (%)**	
Infarcted compared with normal myocardium	344.9 ± 60.4
Blood compared with normal myocardium	144.6 ± 77.3

Mean (± SEM) (*p < 0.05 viable vs infarcted myocardium).

The mean relative SI of infarcted compared with viable myocardium was determined at differing TI values; where TI_-1_, TI_+1_, TI_+2 _and TI_+2 _are 20 msec intervals around TI_0 _(Figure 4). The mean relative SIs (%) of the infarcted myocardium compared with viable myocardium at TI_0 _(344.9 ± 60.4%) was significantly different (p < 0.05) using the Bonferroni test at TI_-1 _(209.3 ± 48.0), at TI_-2 _(190.2 ± 31.8), at TI_+1 _(269.8 ± 47.3) and at TI_+2 _(211.9 ± 46.3).

## Discussion

For both technical and economic reasons, the mouse is currently the most commonly used mammal for studying whole body function and dysfunction. It is therefore important to develop tools that allow the study of mouse models that recapitulate the human disease process under investigation. For cardiac diseases, a range of physiological measures can be used to assess heart morphology and function (echocardiography, conductance volumetry, sonomicrometry and magnetic resonance imaging), pressure, flow, and electrophysiology [8]. Here we report on the feasibility of using high-field MRI to delineate myocardial infarction, with late gadolinium enhancement and an inversion recovery pulse sequence in mice, despite their rapid heart rate (~500 bpm). To our knowledge, this is the first study to demonstrate that an inversion recovery pulse sequence can be used to discriminate infarcted from non-infarcted myocardium in small rodents at 9.4T.

The implementation of an inversion recovery T1-weighted sequence, where the inversion time (TI) is selected to null signal intensity (SI) from the non-infarcted myocardium after the administration of a contrast agent, has improved the contemporary diagnosis of myocardial infarction, and this approach is now used commonly in the clinic [1,9-11]. The main advantage of using an inversion recovery pulse sequence for late gadolinium enhancement MRI is its ability to accurately identify both viable and non-viable myocardium post-infarction. Indeed, hyper-enhancement occurs in the non-viable myocardium due the passive diffusion of gadolinium into the intracellular space because of dysfunctional myocyte membranes [12]. The combination of IR pulse sequence and administration of contrast agent, allows a clear and highly reproducible delineation of hyper-enhanced regions corresponding to the infarcted, non-viable myocardial tissues. Indeed, the post-infarction discrimination between viable and non-viable myocardium, is critical in monitoring prognosis and therapeutic interventions [13]. Moreover, similar studies on large animal models have confirmed the strong correlation between infarct size, as visualised by administration of contrast agent together with use of IR pulse sequence, and subsequent post-mortem analysis [3,14].

Our study demonstrates that the TI, which nulls the signal in the non-infarcted myocardium, was 268 msec ± 27.3 (ranged from 220 to 300 msec). Although this value was obtained at high magnetic field strengths, which results in increased longitudinal relaxation times, the optimal TI is nevertheless comparable to that of many clinical studies. Indeed, Gupta *et al *[2] reported an optimal TI of 260.6 msec ± 38.4 (ranged from 180 to 315 msec) in human subjects, following a bolus injection of gadolinium (0.15 mmol/kg) at 1.5T. The optimized inversion time for myocardial infarction imaging in rabbits was found to be between 180 and 240 msec following 0.1 mmol/kg of Gd-DTPA at 1.5T [15]. Since the high magnetic field leads to an increase of the longitudinal relaxation times, it was anticipated that the TI values in our study would to be higher. Hence, our results may be explained by the effect of 9.4T on the high dose of gadolinium, inducing a larger decrease in T1 than expected. Li *et al *[16] reported a rapid decrease, by half, in T1 values of normal pig myocardium following a Gd-DTPA dose of 0.05 mmol/kg at 7T. In the present study, a gadolinium dose of 0.6 mmol/kg was used, which is in the range reported for previous animal studies [4], but which may have induced a more significant decrease in T1 values than predicted. However, when compared to Schneider *et al*'s T1 values of 1.1 ± 0.3 seconds at 11.7T for the viable left ventricle myocardium in mice [17] and Li *et al*'s observations following Gd-DTPA injection, our study performed at 9.4T yielded relatively low TI values, and are thus reasonable. Similarly, Wagner *et al *[3] reported that at 1.5T, the inversion times selected to null the signal in viable myocardium were higher for a contrast agent dose of 0.1 mmol/kg than for 0.2 mmol/kg. Moreover, the TI could change over time following gadolinium injection, predominantly due to a decrease in concentration of the contrast agent in myocardial tissues [3,6,12]. In our study, the signal intensities in the blood and in the infarcted myocardium were less discriminating, leading to difficulty in visual delineation of the two territories. This was probably due to the relatively high dose of the contrast agent, and the delay between injection and initiation of the imaging acquisition [1]. In retrospect, it would have been helpful to wait longer for the contrast agent to wash out of the blood pool; nevertheless, we found the optimal TI (~270 ms) at a post-injection delay of about 28 minutes.

The higher heart rate in rodents demands an ultrafast imaging protocol to minimize motion artefacts. This rapid heart rate also made it difficult to use cardiac triggering, and moreover, the TR was not fixed, which varied from ~100 ms to 420 ms. It is also important to note that the maximum TI (420 ms) corresponded to ~4 R-R intervals, which may have influenced the CNR values between different myocardial areas. Thus, the contrast observed between the blood, the infarcted and the viable myocardium in the T1-weighted late gadolinium enhanced image, depends on multiple variables such as contrast agent concentration, delay between gadolinium-chelate injection and MR acquisition, clearance rate, and imaging parameters. Blood velocity may also have a role in the contrast, which explains our low CNR between infarcted myocardium and blood [18]. Nevertheless, the present protocol allows the acquisition of workable images in less than 3 minutes, and also allows total TI mapping in less than 45 minutes.

Although respiratory gating may be used to minimize motion related-artefacts, especially at high magnetic fields [17], this approach was not used in our study as respiratory induced motion artefacts were not visible, and therefore had no affect on the definition and contrast across the images. Furthermore, we used serial averaging (20 signal averages) to reduce any residual respiratory artefacts. Moreover, previous studies have also commented on presence of negligible artefacts in mouse heart imaging due to respiratory motion. It was argued that the ratio of breathing to heart rate was lower (1:8) for anesthetized mice, relative to either larger animals or humans [19]. Although, isoflurane, the anaesthetic of choice used in these studies, depresses the overall heart rate, it nevertheless maintains physiologic heart rates and stable heart function [20,21].

In conclusion, we have demonstrated the feasibility of performing late gadolinium enhancement using an IR sequence in mice at 9.4T. Our approach leads to a high CNR between infarcted and non-infarcted myocardium, using a protocol comparable to that used for clinical MR diagnosis of myocardial infarction. Hence, with the increasing use pre-clinical models to investigate cardiac dysfunction, the implementation of such methodologies are a prerequisite for the accurate delineation between infarcted and non-infarcted myocardium in small animals, in order to allow further development of therapeutic interventions.

## Competing interests

The author(s) declare that they have no competing interests.

## Authors' contributions

CC participated in the conception, design, analysis, interpretation of data and drafting of the manuscript; AHH participated in the conception, design and revising of the manuscript; KKB (corresponding author): Design and coordination, interpretation of data, revising of the manuscript and final approval of the manuscript submitted. All authors read and approved the final manuscript.
